# Preparing Dental Hygiene Students to Care for Patients With Breast Cancer

**DOI:** 10.1002/jdd.13999

**Published:** 2025-07-16

**Authors:** Denise M. Messina, Matthew J. Messina

**Affiliations:** ^1^ Division of Dental Hygiene The Ohio State University College of Dentistry Columbus Ohio USA; ^2^ Department of Restorative & Prosthetic Dentistry The Ohio State University College of Dentistry Upper Arlington Ohio USA

## 1 Problem

Breast cancer is the most common cancer in women, with the diagnosis of 30% of all female cancers each year. There are projected to be 316,950 new cases of invasive breast cancer diagnosed in 2025 [1]. Due to increases in treatment success, patients are living longer. Cancer is now considered a chronic disease, requiring a multidisciplinary team approach to patient care [2].

The acute side effects of breast cancer therapies and long‐term complications can have a marked negative impact on oral health related quality of life [3], including oral/pharyngeal mucositis, pain, xerostomia, dental caries, potential opportunistic infections and risk for medication‐related osteonecrosis of the jaw (MRONJ) and periodontal tissue changes [4].

Dental hygienists (DH) often serve as primary oral health care providers. The National Institute of Dental and Craniofacial Research (NIDCR) indicates that oral evaluation is necessary prior to therapy to identify dental needs that could increase the risk or severity of oral complications during cancer treatment [5].

Taichman et al. concluded that dental hygienists lack knowledge concerning the oral health related effects of common drugs used in breast cancer treatment and that it is important to develop interdisciplinary educational programs in DH training [5].

## 2 Solution

In the curriculum for baccalaureate dental hygiene students at The Ohio State University College of Dentistry, the course “Care for Patients with Special Needs” includes lectures recognizing special conditions that require modifications to dental treatment. There is a focus on understanding the oral side effects associated with cancer therapies and recommendations for the patient's oral care. Because breast cancer therapy is the most likely cancer treatment that dental hygienists will encounter in general practice, the authors’ investigation focused on breast cancer therapy and its oral effects.

The College of Dentistry at The Ohio State University presents the unique opportunity for intraprofessional and interprofessional education from the creation of an interdisciplinary clinic that provides a dental home for cancer patients. The Dental Oncology clinic provides care for patients undergoing treatment for breast cancer, prostate cancer, lung cancer, as well as patients undergoing bone marrow transplant procedures for hematologic cancers. The clinic additionally treats patients undergoing head and neck radiation therapy and surgery for oropharyngeal cancers. Second‐year DH students rotate in the Dental Oncology clinic during their specialty clinical rotations.

## 3 Results

A combination of case‐based learning was provided by the Dental Hygiene Course Director and the Director of the Dental Oncology. A 10‐question multiple choice answer pre‐test/post‐test assessment demonstrated that the DH students successfully increased their knowledge about oral side effects of breast cancer treatment, the drugs used in treating breast cancer, and the locations where breast cancer commonly will metastasize. Students dramatically increased their awareness of the significant pre‐cancer treatment oral recommendations for cancer patients, which will improve the level of patient care provided to these at‐risk populations. (Table 1)

**TABLE 1:** Comparison of pre/post‐test results.

Questions:	# of students who correctly identified the answer Pre‐test *N* = 34	# of students who correctly identified the answer Post‐test *N* = 34	Net increase in correct answers from pre‐test to post‐test
What are the oral side effects of breast cancer treatment?	5 (17.6%)	29 (85.3%)	24 (70.5%)
Which class of drugs are used to treat breast cancer?	5 (14.7%)	29 (85.3%)	24 (70.5%)
Metastases of breast cancer often occurs in which locations?	7 (20.5%)	29 (85.3%)	22 (64.7%)
The patient's medication list will indicate scope of cancer treatment. T/F	5 (14.7%)	14 (41.2%)	9 (26.4%)
Which of the following is recommended dental care prior to cancer treatment?	0 (0%)	31 (91.2%)	31 (91.2%)

It remains important to reinforce the importance of taking a thorough medical history, as cancer patients will often forget to mention infusions or injections. The DH should ask questions specifically to reveal the treatment regimen and/or obtain medical consultation with the patient's oncologist to determine the complete picture of the cancer care plan. This allows the development of an appropriate oral health care plan for individual patients.

## Conflicts of Interest

The authors represent that they have no conflicts of interest to disclose.
